# Draft Genome Sequence of the Antimycin-Producing Bacterium *Streptomyces* sp. Strain SM8, Isolated from the Marine Sponge *Haliclona simulans*

**DOI:** 10.1128/genomeA.01535-17

**Published:** 2018-01-25

**Authors:** Eduardo L. Almeida, Lekha M. Margassery, Jonathan Kennedy, Alan D. W. Dobson

**Affiliations:** a School of Microbiology, University College Cork, Cork, Ireland; b Environmental Research Institute, University College Cork, Cork, Ireland

## Abstract

*Streptomyces* sp. strain SM8, isolated from *Haliclona simulans*, possesses antifungal and antibacterial activities and inhibits the calcineurin pathway in yeast. The draft genome sequence is 7,145,211 bp, containing 5,929 predicted coding sequences. Several secondary metabolite biosynthetic gene clusters are present, encoding known and novel metabolites, including antimycin.

## GENOME ANNOUNCEMENT

Marine organisms are a rich source of novel secondary metabolites, with over 1,000 novel marine compounds discovered in 2015 alone (1). Sponges are a significant source of secondary metabolites, and more than 190 different metabolites have been isolated from the genus *Haliclona* alone (2). Many of the metabolites isolated from marine sponges are believed to be of microbial origin, suggesting symbiotic relationships between sponges and associated bioactive-producing microorganisms (3).

In a study of bacteria isolated from the marine sponge *Haliclona simulans* collected from the west coast of Ireland, up to 50% of bacteria were found to produce antibiotic activity against medically important pathogens, such as methicillin-resistant *Staphylococcus aureus* (MRSA) (4). In an initial screen, the *Streptomyces* sp. strain SM8 showed antibacterial and strong antifungal activities (4). Nuclear magnetic resonance (NMR) analysis of the active fractions proved that hydroxylated saturated fatty acids were the major components present in the antibacterial fractions (5). Subsequent screening showed that this strain also produced compounds that inhibited the calcineurin pathway in *Saccharomyces cerevisiae* (6). Metabolic profiling of the compounds produced by the organism identified antimycin as one of the main products with antifungal activity (5). The genome sequence of strain SM8 was determined to facilitate the identification of the range of bioactive compounds produced by the organism and of further heterologous expression of gene clusters encoding products of interest using the transformation-association recombination (TAR) cloning technique (7, 8).

Genomic DNA (gDNA) was obtained as previously described (5). The nucleotide sequence was generated from a fragment library using the GS FLX Titanium system (Roche), resulting in 229,280 reads and 94,668,678 bp. The assembly of the contigs was performed using i) GS De Novo Assembler v2.3 (Roche) software for the *de novo* assembly of reads and then ii) MeDuSa software v1.6 for the reference-based assembly of scaffolds (9), using as the reference the top 3 complete genomes that shared the highest 16S rRNA gene sequence similarities in NCBI’s GenBank database, namely, *Streptomyces sampsonii* strain KJ40 (GenBank accession no. NZ_CP016824), *Streptomyces albus* strain SM254 (NZ_CP014485), and *S. albus* strain J1074 (NC_020990) (10–13). The quality of the final assembly was evaluated with QUAST v4.5 and checkM software, resulting in 11 scaffolds of >500 bp, a length of 7,145,211 bp, a G+C content of 73.33%, and an estimated genome completion of 96.11% (14, 15). The draft sequence was annotated using the NCBI Prokaryotic Genome Annotation Pipeline (PGAP) v4.3, which predicted 5,929 coding sequences (CDSs), 3 rRNAs, and 61 tRNAs (16).

Analysis for the prediction of secondary metabolite gene clusters using antiSMASH v4.0.2 identified several gene clusters encoding polyketide synthases (PKS), nonribosomal peptide synthetases (NRPS), PKS/NRPS hybrids, terpene biosynthesis, and siderophores (17). The complete gene cluster for the biosynthesis of antimycin—a compound which was previously identified by mass spectrometry analysis (5)—was also identified using antiSMASH and manually curated. Knowledge of the genetic basis of secondary metabolism in *Streptomyces* sp. strain SM8 will lead to further characterization of the compounds responsible for the wide range of biological activities present.

### Accession number(s).

This whole-genome shotgun project has been deposited in DDBJ/ENA/GenBank under the accession no. AMPN00000000. The version described in this paper is the second version, AMPN02000000.
